# ADAM-multi: software to simulate complex breeding programs for animals and plants with different ploidy levels and generalized genotypic effect models to account for multiple alleles

**DOI:** 10.3389/fgene.2025.1513615

**Published:** 2025-02-10

**Authors:** Thinh Tuan Chu, Just Jensen

**Affiliations:** ^1^ Center for Quantitative Genetics and Genomics, Aarhus University, Aarhus, Denmark; ^2^ Faculty of Animal Science, Vietnam National University of Agriculture, Hanoi, Vietnam

**Keywords:** stochastic breeding program, genotypic model, polyploidy, dominance, epistasis

## Abstract

Stochastic simulation software, ADAM, has been developed for the purpose of breeding optimization in animals and plants, and for validation of statistical models used in genetic evaluations. Just like other common simulation programs, ADAM assumed the bi-allelic state of quantitative trait locus (QTL). While the bi-allelic state of marker loci is due to the common choice of genotyping technology of single nucleotide polymorphism (SNP) chip, the assumption may not hold for the linked QTL. In the version of ADAM-Multi, we employ a novel simulation model capable of simulating additive, dominance, and epistatic genotypic effects for species with different levels of ploidy, providing with a more realistic assumption of multiple allelism for QTL variants. When assuming bi-allelic QTL, our proposed model becomes identical to the model assumption in common simulation programs, and in genetic textbooks. Along with the description of the updated simulation model in ADAM-Multi, this paper shows two small-scale studies that investigate the effects of multi-allelic versus bi-allelic assumptions in simulation and the use of different prediction models in a single-population breeding program for potatoes. We found that genomic models using dense bi-allelic markers could effectively predicted breeding values of individuals in a well-structure population despite the presence of multi-allelic QTL. Additionally, the small-scale study indicated that including non-additive genetic effects in the prediction model for selection did not lead to an improvement in the rate of genetic gains of the breeding program.

## 1 Introduction

Stochastic simulation is a cost-effective and powerful tool to optimize breeding programs with reduced experimental costs. Such a tool unlocks possibilities for investigating alternative breeding schemes, in order to maximizing genetic gains of the breeding program at a given input of resources. Software package, ADAM (Pedersen et al., 2009; Liu et al., 2019), has been developed for the purpose of breeding optimization in pig, cattle, fish and plants (Bengtsson et al., 2022; Tessema et al., 2020; Zaalberg et al., 2022; Chu et al., 2020). The tool is also very useful in validating statistical genetic prediction models (Romé et al., 2023; Chu et al., 2021) and in studying methods for preserving genetic diversity in breeding programs (Henryon et al., 2015). Over time the software has been further developed and updated with many features including extension to non-additive genetic models (Chu et al., 2024), indirect genetic effects (Chu et al., 2021), categorical traits (Gebreyesus et al., 2020), definition of true inbreeding (Henryon et al., 2019), or extension to plant breeding (Liu et al., 2019).

ADAM (Pedersen et al., 2009) simulates genotypic effects for each allele in a quantitative trait locus (QTL). This model is unique from other software like AlphaSim (Gaynor et al., 2021; Faux et al., 2016), ChromaX (Younis et al., 2023), MoBPS (Pook et al., 2020), SeqBreed (Pérez-Enciso et al., 2020) and XSim (Chen et al., 2022) that use substitution genotypic effects of QTL. However, all of these tools including ADAM assume a bi-allelic state of each segregating QTL. This assumption might come from current, common genetic models (Christensen et al., 2012; VanRaden, 2008; Falconer and Mackay, 1996) that assume substitution effects of one allele to its alternative. While the bi-allelic state of marker loci is due to the common choice of genotyping technology that yield single nucleotide polymorphism (SNP). The assumption of bi-allelic QTL may not true for all QTL. Multiple alleles have been shown in numerous QTL (Biová et al., 2024; Jiang et al., 2020). In addition, multi-allelic models of QTL are more reasonable explanations for different functional genetic effects from unrelated populations (González-Diéguez et al., 2021).

Multi-allelic models have been developed for genomic prediction in diploid species (Álvarez-Castro and Crujeiras, 2019; Álvarez-Castro and Yang, 2011; Yang and Álvarez-Castro, 2008; Da, 2015). Relevant model based on haplotype blocks also have been shown for genomic prediction (Weber et al., 2023). Thérèse Navarro et al. (2022) has developed a package for genome wide association studies (GWAS) of polyploid populations with multi-allelic models, but only the additive genetic effects were included in the model. For the purpose of simulation, however, we are not aware of any studies that have used multi-allelic models with additive, dominance and epistatic genetic effects, or accounting for different levels of ploidy.

In addition to bi-allelic assumptions, earlier version of ADAM (Pedersen et al., 2009; Liu et al., 2019) use was limited to diploid species only. Many economically important species like potato, banana, sugar cane and some fish orders of salmonids and common carps are polyploid. Extension of simulation models to different ploidy levels is necessary for ADAM (Pedersen et al., 2009; Liu et al., 2019) to design complex breeding schemes for these species.

This paper will describe new features of software package, now called ADAM-Multi, for simulating breeding programs for plants and animals. The focus will be on description of genotypic models for simulating traits with genotypic effects of additive, dominance, and epistatic genetics for species with different ploidy levels including extensions to multi-allelic assumptions. The methods implemented are illustrated in two examples that study the effects of different assumptions on number of alleles, ploidy level and different prediction models used in selection.

## 2 Materials and methods

### 2.1 Genotypic models for simulation

We aim to simulate genotypic effects that are generalized to multi-allelic QTL with number ( $n_{B}$ ) of alleles, and the genome with ploidy level of $n_{ploidy}$ . Assuming a QTL with alleles $B_{1}$ , $B_{2}$ ,…, $B_{n_{B}}$ , ADAM-Multi uses following model to simulate additive genotypic value ( a ) at one locus:

$$a=\sum_{i_{B}}^{n_{B}}t_{i_{B}}^{a}a_{i_{B}}=t_{1}^{a}a_{1}+t_{2}^{a}a_{2}+\ldots +t_{n_{B}}^{a}a_{n_{B}}$$ (1)

where a is the additive genotypic value of a QTL; $a_{i_{B}}$ is genotypic additive effect of allele $B_{i_{B}}$ (or called $i_{B}$ for short) at the QTL; $t_{i_{B}}^{a}$ is the additive covariate for allele $i_{B}$ that is scaled genotype dosage calculated as in the AlphaSimR software (Gaynor et al., 2021):

$$t_{i_{B}}^{a}=\left(t_{i_{B}}-\frac{n_{ploidy}}{2}\right)\left(\frac{2}{n_{ploidy}}\right)$$ (2)

where $t_{i_{B}}$ is a raw genotype dosage, or number of copies of allele $i_{B}$ at the locus, $n_{ploidy}$ is the ploidy level of genome. Key notations are defined in Table 1.

**TABLE 1:** List of key symbols.

Symbol	Definition
Simulation models at one-locus level	
$n_{B}$	Defined number of alleles in a QTL. It is also the maximum number of segregating alleles for all QTL.
$B_{1}$ , $B_{2}$ , $B_{i_{B}}$ ,…, $B_{n_{B}}$	Allele 1st, 2nd, $i_{B}$ ^th^, … , $n_{B}$ ^th^ of B. Allele $i_{B}$ is referred to $B_{i_{B}}$
$t_{1}$ , $t_{2}$ , $t_{i_{B}}$ ,…, $t_{n_{B}}$	A raw genotype dosage, or number of copies of allele $B_{1}$ , $B_{2}$ , $B_{i_{B}}$ ,…, $B_{n_{B}}$ at the locus
$n_{ploidy}$	Ploidy level of genome
$t_{1}^{a}$ , $t_{2}^{a}$ , $t_{i_{B}}^{a}$ ,…, $t_{n_{B}}^{a}$	Additive effect covariates, or scaled genotype dosages for additive genetic effect corresponding to $t_{1}$ , $t_{2}$ , $t_{i_{B}}$ ,…, $t_{n_{B}}$ at the locus for allele $B_{1}$ , $B_{2}$ , $B_{i_{B}}$ ,…, $B_{n_{B}}$
$a_{1}$ , $a_{2}$ , $a_{i_{B}}$ ,…, $a_{n_{B}}$	Additive genotypic effect of allele $B_{1}$ , $B_{2}$ , $B_{i_{B}}$ ,…, $B_{n_{B}}$ at a locus
$t_{1}^{d}$ , $t_{2}^{d}$ , $t_{i_{B}}^{d}$ ,…, $t_{n_{B}}^{d}$	Dominance effect covariates, or scaled genotype dosages for dominance effect corresponding to $t_{1}$ , $t_{2}$ , $t_{i_{B}}$ ,…, $t_{n_{B}}$ at the locus for allele $B_{1}$ , $B_{2}$ , $B_{i_{B}}$ ,…, $B_{n_{B}}$
$d_{1}$ , $d_{2}$ , $d_{i_{B}}$ ,…, $d_{n_{B}}$	Dominance genotypic effect of allele $B_{1}$ , $B_{2}$ , $B_{i_{B}}$ ,…, $B_{n_{B}}$ at the locus
a , d	Additive, dominance genotypic value at the locus level
Simulation models of two-loci epistatic interactions	
kl	The epistatic interaction pair between loci k and l
$t_{i_{B}}^{a,x}$ , $t_{i_{B}}^{d,x}$	Additive, and dominance covariates for the effect of allele $i_{B}$ ^th^ at locus x . Here, x represents k and l in two-loci epistasis
${\left(aa\right)}^{kl}$ , ${\left(dd\right)}^{kl}$	Additive × additive, and dominance × dominance genotypic values of the epistatic interaction between the pair of loci k and l
${\left(ad\right)}^{kl}$	Additive-dominance genotypic values of the epistatic interaction that includes both additive × dominance and dominance × additive between the pair of loci k and l
${\left(aa\right)}_{i_{B}^{kl}}^{kl}$ , ${\left(ad\right)}_{i_{B}^{kl}}^{kl}$ , ${\left(da\right)}_{i_{B}^{kl}}^{kl}$ , ${\left(dd\right)}_{i_{B}^{kl}}^{kl}$	Additive × additive, additive × dominance, dominance × additive, dominance × dominance genotypic effects of the epistatic interaction between allele $i_{B}^{k}$ of locus k and allele $i_{B}^{l}$ of locus l
Simulation models at individual level	
$g_{i}$	Total genetic value of individual i
$n_{qtl}$ , $n_{ep}$	Number of QTL, and number of epistatic interactions
a , d	$n_{B}\times n_{qtl}$ matrices of additive and dominance genotypic effects of $n_{B}$ alleles for $n_{qtl}$ QTL
$t_{i}^{a}$ , $t_{i}^{d}$	$n_{qtl}\times n_{B}$ matrices of additive and dominance covariates of $n_{B}$ alleles for $n_{qtl}$ QTL of individual i
$\left(\text{aa}\right)$ , $\left(\text{ad}\right)$ , $\left(\text{da}\right)$ , $\left(\text{dd}\right)$	Matrices of additive × additive, additive × dominance, dominance × additive, dominance × dominance epistatic effects with a dimension of $\left(n_{B}\times n_{B}\right)$ rows and $n_{ep}$ columns
$t_{i}^{\text{aa}}$ , $t_{i}^{\text{ad}}$ , $t_{i}^{\text{da}}$ , $t_{i}^{\text{dd}}$	Matrices of additive × additive, additive × dominance, dominance × additive, dominance × dominance epistatic covariates with a dimension of $n_{ep}$ rows and $\left(n_{B}\times n_{B}\right)$ columns for individual i
$a^{*}$ , $d^{*}$ , ${\left(\text{aa}\right)}^{*}$ , ${\left(\text{ad}\right)}^{*}$ , ${\left(\text{da}\right)}^{*}$ , ${\left(\text{dd}\right)}^{*}$	Starting (prior) values of a , d **,** $\left(\text{aa}\right)$ , $\left(\text{ad}\right)$ , $\left(\text{da}\right)$ and $\left(\text{dd}\right)$
$\sigma_{A}^{2}$ , $\sigma_{D}^{2}$ , $\sigma_{AA}^{2}$ , $\sigma_{AD}^{2}$ , $\sigma_{DD}^{2}$	Functional variances of additive, dominance, additive × additive, additive-dominance, and dominance × dominance for simulation
Prediction models based on population level	
u , v , $\left(\text{uu}\right)$ , $\left(\text{uv}\right)$ , $\left(\text{vv}\right)$	Vectors of individuals’ additive, dominance, and epistatic effects
$G_{u}$ , $G_{v}$ , $G_{\text{uu}}$ , $G_{\text{uv}}$ , $G_{\text{vv}}$	Genomic relationship matrices constructed based on bi-allelic markers
$\sigma_{u}^{2}$ , $\sigma_{v}^{2}$ , $\sigma_{uu}^{2}$ , $\sigma_{uv}^{2}$ , $\sigma_{vv}^{2}$	Statistical variances of additive, dominance, additive × additive, additive-dominance, and dominance × dominance

Similarly, the genotypic model for dominance value ( d ) of a QTL at the locus level is:

$$d=\sum_{i_{B}}^{n_{B}}t_{i_{B}}^{d}d_{i_{B}}=t_{1}^{d}d_{1}+t_{2}^{d}d_{2}+\ldots +t_{n_{B}}^{d}d_{n_{B}}$$ (3)

where $d_{i_{B}}$ is dominance genotypic effect of allele $i_{B};\;t_{i_{B}}^{d}$ is the dominance covariate for allele $i_{B}$ that is scaled genotype dosage calculated as in AlphaSimR (Gaynor et al., 2021):

$$t_{i_{B}}^{d}=t_{i_{B}}\left(n_{ploidy}-t_{i_{B}}\right){\left(\frac{2}{n_{ploidy}}\right)}^{2}$$ (4)

This simulation model assumes digenic dominance for each allele, i.e., each allele has the same dominance effect with all other alleles. Table 2 shows examples of t , $t^{a}$ and $t^{d}$ for diploid and tetraploid genome assuming $n_{B}=2$ with allele $B_{1}$ and $B_{2}$ .

**TABLE 2:** Conversion from raw genotype dosages ( $t_{1}$ , $t_{2}$ ) to additive ( $t_{1}^{a}$ , $t_{2}^{a}$ ) and dominance ( $t_{1}^{d}$ , $t_{2}^{d}$ ) covariates when assuming bi-allelic loci.

Genotype	$t_{1}$	$t_{2}$	$t_{1}^{a}$	$t_{2}^{a}$	$t_{1}^{d}$	$t_{2}^{d}$
Diploid						
$B_{1}B_{1}$	2	0	1	−1	0	0
$B_{1}B_{2}$	1	1	0	0	1	1
$B_{2}B_{2}$	0	2	−1	1	0	0
Tetraploid						
$B_{1}B_{1}B_{1}B_{1}$	4	0	1	−1	0	0
$B_{1}B_{1}B_{1}B_{2}$	3	1	$\frac{1}{2}$	$-\frac{1}{2}$	$\frac{3}{4}$	$\frac{3}{4}$
$B_{1}B_{1}B_{2}B_{2}$	2	2	0	0	1	1
$B_{1}B_{2}B_{2}B_{2}$	1	3	$-\frac{1}{2}$	$\frac{1}{2}$	$\frac{3}{4}$	$\frac{3}{4}$
$B_{2}B_{2}B_{2}B_{2}$	0	4	−1	1	0	0

The simulation model for additive × additive genotypic value ${\left(aa\right)}^{kl}$ of the two-locus epistatic interaction between the pair of loci k and l :

$${\left(aa\right)}^{kl}=\left\{\left[\begin{array}{cccc} t_{1}^{a,l} & t_{2}^{a,l} & \ldots & t_{n_{B}}^{a,l} \end{array}\right]⊗\left[\begin{array}{cccc} t_{1}^{a,k} & t_{2}^{a,k} & \ldots & t_{n_{B}}^{a,k} \end{array}\right]\right\}\cdot \left[\begin{array}{c} {\left(aa\right)}_{1}^{kl}\\ {\left(aa\right)}_{2}^{kl}\\ \ldots \\ {\left(aa\right)}_{n_{B}\times n_{B}}^{kl} \end{array}\right]$$ (5)

where $\left[\begin{array}{cccc} t_{1}^{a,x} & t_{2}^{a,x} & \ldots & t_{n_{B}}^{a,x} \end{array}\right]$ is a vector of additive covariates for locus x ( k or l ) with $n_{B}$ elements; ⊗ denotes the Kronecker product; denotes the symbol for matrix multiplication; $\left[\begin{array}{c} {\left(aa\right)}_{1}^{kl}\\ {\left(aa\right)}_{2}^{kl}\\ \ldots \\ {\left(aa\right)}_{n_{B}\times n_{B}}^{kl} \end{array}\right]$ is vector of additive × additive genotypic effects that have $n_{B}\times n_{B}$ elements. In Equation 5, the number of alleles in loci k and l are the same, and equal to $n_{B}$ . Simulation models in this paper consider a fixed number $n_{B}$ for all QTL, even if not all alleles in a QTL are segregating. Genotypic models in case of arbitrary number of alleles for QTL that set the effects of non-segregating alleles to zero can be found in Supplementary Appendix 1.

The simulation model for additive-dominance genotypic value ${\left(ad\right)}^{kl}$ of the epistatic interaction is the sum of additive × dominance and dominance × additive interaction between the pair of loci k and l :

$${\left(ad\right)}^{kl}=\left\{\left[\begin{array}{cccc} t_{1}^{d,l} & t_{2}^{d,l} & \ldots & t_{n_{B}}^{d,l} \end{array}\right]⊗\left[\begin{array}{cccc} t_{1}^{a,k} & t_{2}^{a,k} & \ldots & t_{n_{B}}^{a,k} \end{array}\right]\right\}\cdot \left[\begin{array}{c} {\left(ad\right)}_{1}^{kl}\\ {\left(ad\right)}_{2}^{kl}\\ \ldots \\ {\left(ad\right)}_{n_{B}\times n_{B}}^{kl} \end{array}\right]+\left\{\left[\begin{array}{cccc} t_{1}^{a,l} & t_{2}^{a,l} & \ldots & t_{n_{B}}^{a,l} \end{array}\right]⊗\left[\begin{array}{cccc} t_{1}^{d,k} & t_{2}^{d,k} & \ldots & t_{n_{B}}^{d,k} \end{array}\right]\right\}\cdot \left[\begin{array}{c} {\left(da\right)}_{1}^{kl}\\ {\left(da\right)}_{2}^{kl}\\ \ldots \\ {\left(da\right)}_{n_{B}\times n_{B}}^{kl} \end{array}\right]$$ (6)

where $\left[\begin{array}{cccc} t_{1}^{d,x} & t_{2}^{d,x} & \ldots & t_{n_{B}}^{d,x} \end{array}\right]$ is a vector of dominance covariates (Equation 4) for locus x with $n_{B}$ elements; $\left[\begin{array}{c} {\left(ad\right)}_{1}^{kl}\\ {\left(ad\right)}_{2}^{kl}\\ \ldots \\ {\left(ad\right)}_{n_{B}\times n_{B}}^{kl} \end{array}\right]\text{ and }\left[\begin{array}{c} {\left(da\right)}_{1}^{kl}\\ {\left(da\right)}_{2}^{kl}\\ \ldots \\ {\left(da\right)}_{n_{B}\times n_{B}}^{kl} \end{array}\right]$ are vectors of additive × dominance ( ${\left(ad\right)}_{x}^{kl}$ ), and dominance × additive ( ${\left(da\right)}_{x}^{kl}$ ) genotypic effects that have $n_{B}\times n_{B}$ elements. The value ${\left(ad\right)}_{x}^{kl}$ is different from ${\left(da\right)}_{x}^{kl}$ .

The simulation model for dominance × dominance genotypic value ${\left(dd\right)}^{kl}$ of the epistatic interaction between the pair of loci k and l :

$${\left(dd\right)}^{kl}=\left\{\left[\begin{array}{cccc} t_{1}^{d,l} & t_{2}^{d,l} & \ldots & t_{n_{B}}^{d,l} \end{array}\right]⊗\left[\begin{array}{cccc} t_{1}^{d,k} & t_{2}^{d,k} & \ldots & t_{n_{B}}^{d,k} \end{array}\right]\right\}\cdot \left[\begin{array}{c} {\left(dd\right)}_{1}^{kl}\\ {\left(dd\right)}_{2}^{kl}\\ \ldots \\ {\left(dd\right)}_{n_{B}\times n_{B}}^{kl} \end{array}\right]$$ (7)

where $\left[\begin{array}{c} {\left(dd\right)}_{1}^{kl}\\ {\left(dd\right)}_{2}^{kl}\\ \ldots \\ {\left(dd\right)}_{n_{B}\times n_{B}}^{kl} \end{array}\right]$ is vector of dominance × dominance genotypic effects that have $n_{B}\times n_{B}$ elements.

So far, the simulated genotypic values were presented at the levels of locus and loci pairs. Here, we present the model for simulating genotypic value at the individual level, which is the sum effects of all QTLs and epistatic pair interactions. The model in a matrix form for total genotypic value $g_{i}$ of individual i is:

$$g_{i}=\text{tr}\left(t_{i}^{a}\cdot a\right)+\text{tr}\left(t_{i}^{d}\cdot d\right)+\text{tr}\left[t_{i}^{\text{aa}}\cdot \left(\text{aa}\right)\right]+\text{tr}\left[t_{i}^{\text{dd}}\cdot \left(\text{dd}\right)\right]+\text{tr}\left[t_{i}^{\text{ad}}\cdot \left(\text{ad}\right)\right]+\text{tr}\left[t_{i}^{\text{da}}\cdot \left(\text{da}\right)\right]$$ (8)

where a is a $n_{B}\times n_{qtl}$ matrix:

$$a=\left[\begin{array}{cccc} a_{1}^{j_{1}} & a_{1}^{j_{2}} & \ldots & a_{1}^{n_{qtl}}\\ a_{2}^{j_{1}} & a_{2}^{j_{2}} & \ldots & a_{2}^{n_{qtl}}\\ \ldots & \ldots & \ldots & \ldots \\ a_{n_{B}}^{j_{1}} & a_{n_{B}}^{j_{2}} & \ldots & a_{n_{B}}^{n_{qtl}} \end{array}\right],$$

where $n_{qtl}$ is the number of $\text{QTL};\;a_{i_{B}}^{j_{qtl}}$ is the additive genotypic effect of allele $i_{B}$ at $\text{QTL }j_{qtl};\;t_{i}^{a}\text{ is }a\;n_{qtl}\times n_{B}$ matrix:

$$t_{i}^{a}=\left[\begin{array}{cccc} t_{1,i}^{a,1} & t_{2,i}^{a,1} & \ldots & t_{n_{B},i}^{a,1}\\ t_{1,i}^{a,2} & t_{2,i}^{a,2} & \ldots & t_{n_{B},i}^{a,2}\\ \ldots & \ldots & \ldots & \ldots \\ t_{1,i}^{a,n_{qtl}} & t_{2,i}^{a,n_{qtl}} & \ldots & t_{n_{B},i}^{a,n_{qtl}} \end{array}\right],$$

where $t_{i_{B},i}^{a,j_{qtl}}$ is the additive covariate of allele $i_{B}$ at $\text{QTL }j_{qtl}$ of individual $i.\;t_{i_{B},i}^{a,j_{qtl}}$ can be calculated based on the genotype of individual i at locus $j_{qtl}$ using Equation 2; $\text{tr}\left(\right)$ is the trace of a matrix. Similarly, d is a $n_{B}\times n_{qtl}$ matrix of dominance genotypic effects; $t_{i}^{d}$ is a $n_{qtl}\times n_{B}$ matrix of dominance covariate of individual i . Matrix $\left(\text{aa}\right)$ has a dimension of $\left(n_{B}\times n_{B}\right)$ rows and $n_{ep}$ columns:

$$\left(\text{aa}\right)=\left[\begin{array}{cccc} {\left(aa\right)}_{1,1}^{kl} & {\left(aa\right)}_{2,1}^{kl} & \ldots & {\left(aa\right)}_{n_{ep},1}^{kl}\\ {\left(aa\right)}_{1,2}^{kl} & {\left(aa\right)}_{2,2}^{kl} & \ldots & {\left(aa\right)}_{n_{ep},2}^{kl}\\ \ldots & \ldots & \ldots & \ldots \\ {\left(aa\right)}_{1,\left(n_{B}\times n_{B}\right)}^{kl} & {\left(aa\right)}_{2,\left(n_{B}\times n_{B}\right)}^{kl} & \ldots & {\left(aa\right)}_{n_{ep},\left(n_{B}\times n_{B}\right)}^{kl} \end{array}\right],$$

where $n_{ep}$ is number of epistatic interactions between pairs of loci; ${\left(aa\right)}_{j_{ep},\left(i_{B}^{k},i_{B}^{l}\right)}^{kl}$ is the epistatic additive × additive effect at the interaction pair $j_{ep}$ between allele $i_{B}^{k}$ of locus k and allele $i_{B}^{l}$ of locus $l.\;t_{i}^{\text{aa}}$ is a matrix with $n_{ep}$ rows and $\left(n_{B}\times n_{B}\right)$ columns.Row $j_{ep}$ th of $t_{i}^{\text{aa}}$ is set up as in Equation 5. For example,

$$t_{j_{\text{ep}},i}^{\text{aa}}=\left[\begin{array}{cccc} t_{j_{ep},1,i}^{a,l} & t_{j_{ep},2,i}^{a,l} & \ldots & t_{j_{ep},n_{B},i}^{a,l} \end{array}\right]⊗\left[\begin{array}{cccc} t_{j_{ep},1,i}^{a,k} & t_{j_{ep},2,i}^{a,k} & \ldots & t_{j_{ep},n_{B},i}^{a,k} \end{array}\right],$$

where $t_{j_{ep},i_{B}^{x},i}^{a,x}$ is the additive covariate of allele $i_{B}^{k}$ of QTL k or allele $i_{B}^{l}$ of QTL l at the interaction pair $j_{ep}$ for individual i .

Similarly, $\left(\text{dd}\right)$ is a $\left(n_{B}\times n_{B}\right)\times n_{ep}$ matrix of dominance × dominance genotypic effects; $t_{i}^{\text{dd}}\text{ is }a\;n_{ep}\times \left(n_{B}\times n_{B}\right)$ matrix of dominance × dominance covariates of individual i . The genotypic additive-dominance effects consist of two components: additive × dominance $\text{tr}\left[t_{i}^{\text{ad}}\cdot \left(\text{ad}\right)\right]$ and dominance × additive $\text{tr}\left[t_{i}^{\text{da}}\cdot \left(\text{da}\right)\right]$ . Similar to $\left(\text{dd}\right)$ , matrices $\left(\text{ad}\right)$ and $\left(\text{da}\right)$ have dimension of $\left(n_{B}\times n_{B}\right)\times n_{ep}$ . $t_{i}^{\text{ad}}$ and $t_{i}^{\text{da}}$ are matrices with $n_{ep}$ rows and $\left(n_{B}\times n_{B}\right)$ columns. Row $j_{ep}$ th of $t_{i}^{\text{ad}}$ is set up as in Equation 6. For example,

$$t_{j_{\text{ep}},i}^{\text{ad}}=\left[\begin{array}{cccc} t_{j_{ep},1,i}^{d,l} & t_{j_{ep},2,i}^{d,l} & \ldots & t_{j_{ep},n_{B},i}^{d,l} \end{array}\right]⊗\left[\begin{array}{cccc} t_{j_{ep},1,i}^{a,k} & t_{j_{ep},2,i}^{a,k} & \ldots & t_{j_{ep},n_{B},i}^{a,k} \end{array}\right],$$

where $t_{j_{ep},i_{B}^{x},i}^{d,x}$ is the dominance covariate of allele $i_{B}^{k}$ of QTL k or allele $i_{B}^{l}$ of QTL l at the interaction pair $j_{ep}$ for individual i . Row $j_{ep}$ th of $t_{i}^{\text{da}}$ is:

$$t_{j_{\text{ep}},i}^{\text{da}}=\left[\begin{array}{cccc} t_{j_{ep},1,i}^{a,l} & t_{j_{ep},2,i}^{a,l} & \ldots & t_{j_{ep},n_{B},i}^{a,l} \end{array}\right]⊗\left[\begin{array}{cccc} t_{j_{ep},1,i}^{d,k} & t_{j_{ep},2,i}^{d,k} & \ldots & t_{j_{ep},n_{B},i}^{d,k} \end{array}\right].$$

### 2.2 Stochastic simulation

Details of simulation steps and theoretical principles of ADAM-Multi can be found in previous version by Pedersen et al. (2009) and Liu et al. (2019). These principles are also similar to those in AphaSim (Gaynor et al., 2021). Simulation of genomic models with ADAM first starts with founder haplotypes of a defined genome structure. To create linkage disequilibrium (LD) between QTL and markers, ADAM-Multi can be used in case of multi-allelism. Other packages such as QMSIM (Sargolzaei and Schenkel, 2009) and AlphaSim (Gaynor et al., 2021) do not support multi-allelic models, but they can be used to generate the genome with a specified degree of LD in case of bi-allelic loci. The genotypic effects of alleles in QTLs are sampled, and then centered and scaled to user-defined parameters using the founders’ QTL haplotypes (Chu et al., 2024). Steps for generating additive effects of alleles a ( $n_{B}\times n_{qtl}$ matrix) in ADAM-Multi are:

- Sampling: Each element of matrix $a^{s}$ is sampled from a user-defined normal distribution, e.g., mean of zero and additive variance $\sigma_{A}^{2}$ [or N(0, $\sigma_{A}^{2}$ )]. Matrix $a^{s}$ with the same dimension as a contains starting values of additive effects.
- Centering: Based on matrix $a^{s}$ and genotypes of a founder population, we can calculate population mean at each QTL locus. Additive effects of alleles within each QTL (each row of $a^{s}$ ) are centered to achieve population mean of zero. For example,: ${a_{i_{B}}^{j_{qtl}}}^{*}=a_{i_{B}}^{j_{qtl^{s}}}-{\mu^{j_{qtl}}}^{s},\text{where }{\mu^{j_{qtl}}}^{s}$ is the population mean at locus $j_{qtl}$ given $a^{s}$ (before being centered) and genotypes of the founder population; ${a_{i_{B}}^{j_{qtl}}}^{*}$ is the prior value after centering. Matrix $a^{*}$ (same dimension as a ) with elements of ${a_{i_{B}}^{j_{qtl}}}^{*}$ is the prior values of additive effects after centering.
- Rescaling: Prior variance $\sigma_{A^{*}}^{2}$ can also be calculated as we know all individuals’ genotype in the population and functional effects of QTL ( $a^{*}$ ). Calculation of the variance can be done by different methods including variance by locus, by chromosome, or by individual as in Chu et al. (2024). Additive effects a are calculated by rescaling prior effects $a^{*}$ to achieve the user-defined variance input $\sigma_{A}^{2}$ for the founder population. For example:
$$a=a^{*}\times \sqrt{\frac{\sigma_{A}^{2}}{\sigma_{A^{*}}^{2}}}$$

The calculated variances of $\sigma_{A}^{2}$ and $\sigma_{A^{*}}^{2}$ in the rescaling step are functional, biological or genotypic parameters, which are different from classical, statistical quantitative parameters. The differences between functional and statistical variances are detailed and explained in Álvarez-Castro and Carlborg (2007), Álvarez-Castro and Yang (2011), Chu et al. (2024), and Vitezica et al. (2017). Functional effects of dominance and epistasis are also generated based on user-defined inputs of functional variances. Steps for generating dominance effects of alleles d ( $n_{B}\times n_{qtl}$ matrix) are:

- Sampling: A dominance degree $\delta_{i_{B}}^{d,j_{qtl}}$ for allele $i_{B}$ of QTL $j_{qtl}$ is sampled from a user-defined normal distribution $N\left(\mu_{\delta },\sigma_{\delta }^{2}\right)$ , where $\mu_{\delta }$ is the mean of dominance degree, and $\sigma_{\delta }^{2}$ is the variance of dominance degree. If a user does not provide this distribution, ADAM-Multi default values are $N\left(0.19,0.097\right)$ as in Wellmann and Bennewitz, (2011). Each element of matrix $d^{*}$ (starting (or prior) values of dominance effects) is generated as:
$${d_{i_{B}}^{j_{qtl}}}^{*}=\delta_{i_{B}}^{d,j_{qtl}}*\left|a_{i_{B}}^{j_{qtl}}\right|.$$
- Rescaling: This is done similarly to rescaling as for simulating additive effects. Based on matrix $d^{*}$ and QTL genotypes of a founder population, prior variance $\sigma_{D^{*}}^{2}$ can be calculated. Dominance effects d are the prior effects $d^{*}$ that are rescaled to achieve the user-defined variance inputs of $\sigma_{D}^{2}$ for the founder population.

Steps for sampling additive × additive effects are.

- Sampling: Each element of matrix ${\left(\text{aa}\right)}^{*}$ [starting (or prior) values of additive × additive effects] is sampled from a user-defined normal distribution N(0, $\sigma_{AA}^{2}$ ).
- Rescaling: Prior variance $\sigma_{{AA}^{*}}^{2}$ can be calculated as we know all individuals’ genotype in the population and functional effects of QTL ${\left(\text{aa}\right)}^{*}$ . Calculation of the variance can be done by different methods including variance by pair loci, or by individual as in Chu et al. (2024). Additive × additive effects $\left(\text{aa}\right)$ are obtained by rescaling prior effects ${\left(\text{aa}\right)}^{*}$ to achieve the desired variance inputs of $\sigma_{AA}^{2}$ for the founder population.

Steps for sampling dominance × dominance effects are similar to those for additive × additive effects. Steps for sampling additive-dominance effects are:

- Sampling: Each element of matrices ${\left(\text{ad}\right)}^{*}$ and ${\left(\text{da}\right)}^{*}$ [starting (or prior) values of additive × dominance and dominance × additive effects] is sampled from a user-defined normal distribution N(0, $\sigma_{AD}^{2}$ ).
- Rescaling: Prior variance $\sigma_{{AD}^{*}}^{2}$ can be calculated as we know all individuals’ genotype in the population and functional effects of QTL ${\left(\text{ad}\right)}^{*}$ and ${\left(\text{da}\right)}^{*}$ . Note that the additive-dominance effects of an individuals’ genotype is the sum of additive × dominance and dominance × additive. Effects $\left(\text{ad}\right)$ and $\left(\text{da}\right)$ are obtained by rescaling prior effects ${\left(\text{ad}\right)}^{*}$ and ${\left(\text{da}\right)}^{*}$ to achieve the desired variance inputs of $\sigma_{AD}^{2}$ for the founder population. For example:
$$\left(\text{ad}\right)={\left(\text{ad}\right)}^{*}\times \sqrt{\frac{\sigma_{AD}^{2}}{\sigma_{{AD}^{*}}^{2}}}\text{ and }\left(\text{da}\right)={\left(\text{da}\right)}^{*}\times \sqrt{\frac{\sigma_{AD}^{2}}{\sigma_{{AD}^{*}}^{2}}}.$$

A centering step is not included for dominance and epistatic effects, but the total genetic value $g_{i}$ is centered to achieve the population mean of zero based on the founder population. For example, the model for simulating individuals’ phenotypes is:

$$y_{i}=\mu +g_{i}+e_{i}$$ (9)

where $g_{i}$ is the genetic values of individual i that is constructed as in (Equation 8); μ is the population mean to re-adjust the mean of the founder population to zero.

In ADAM-Multi, model (Equation 9) can be extended to repeated records, inclusion of non-genetic effects, and multiple traits. The functional genetic effects of additive, dominance, and epistasis are independent of allele frequencies, and in the simulation, they are kept constant across generations. Modeling of genetic recombination during meiosis uses bivalent chromosome pairing (Voorrips and Maliepaard, 2012). A breeding scheme is simulated by combining series of actions: mating, reproduction, phenotyping, genotyping, prediction of breeding values and different selection methods. The use of ADAM-Multi is demonstrated in two examples that study the effects of multi-allelic versus bi-allelic assumptions and the use of different prediction models on accuracy of prediction and genetic gains of breeding programs for potato.

### 2.3 Example 1

The example uses a simulation model with additive effects only for a single-population breeding scheme. The investigated factors include different multi-allelic assumptions (6 levels) and two levels of ploidy (Table 3). In total, there were 6 × 2 = 12 scenarios simulated in example 1.

**TABLE 3:** Overview of factors investigated in example 1 and 2.

Factor	Levels
Example 1	
Simulation model for individuals’ genetic values	Additive genetic effect only
Multi-allelic assumption with percentage of QTL having bi-allelic state (6 levels)	Bi-allele (100%)
	Bi-allele (80%) + tri-allele (20%)
	Bi-allele (50%) + tri-allele (50%)
	Bi-allele (80%) + quad-allele (20%)
	Bi-allele (50%) + quad-allele (50%)
	Bi-allele (20%) + quad-allele (80%)
Genetic effects included in prediction model for selection (1 level)	Additive genetic effect only
Ploidy (2 levels)	Diploids
	Tetraploids
Example 2	
Simulation model for individuals’ genetic values	Additive, dominance, additive × additive, additive-dominance, and dominance × dominance effects
Multi-allelic assumption with percentage of QTL having bi-allelic state (2 levels)	Bi-allele (100%)
	Bi-allele (20%) + quad-allele (80%)
Genetic effects included in prediction model for selection (3 levels)	Additive only
	Additive and dominance
	Additive, dominance, epistatic effects
Ploidy (2 levels)	Diploids
	Tetraploids

The simulation model for individuals’ phenotype in example 1 was:

$$y_{i}=\mu +\sum_{i_{B}}^{n_{B}}t_{i_{B},i}^{a,1}a_{i_{B}}^{j_{1}}+\sum_{i_{B}}^{n_{B}}t_{i_{B},i}^{a,2}a_{i_{B}}^{j_{2}}+\ldots +\sum_{i_{B}}^{n_{B}}t_{i_{B},i}^{a,n_{qtl}}a_{i_{B}}^{n_{qtl}}+e_{i}$$ (10)

Simulation of genetic values in (Equation 10) is an extension of (Equation 1) to the sum effects of $n_{qtl}$ , or equivalent to the additive part of (Equation 8). Notations and symbols are the same as in (Equation 8). The environment term $e_{i}$ was drawn from a normal distribution N(0, $\sigma_{e}^{2}$ ) with $\sigma_{e}^{2}=2$ . This example used a simplified simulation model as compared to Equation 9 because we would like to assess accuracy of predicted breeding values in selection. When non-additive genetics are included in the model, the definition of accuracy of predicted breeding values with multi-allelic assumption is unclear in literature.

**FIGURE 1:**
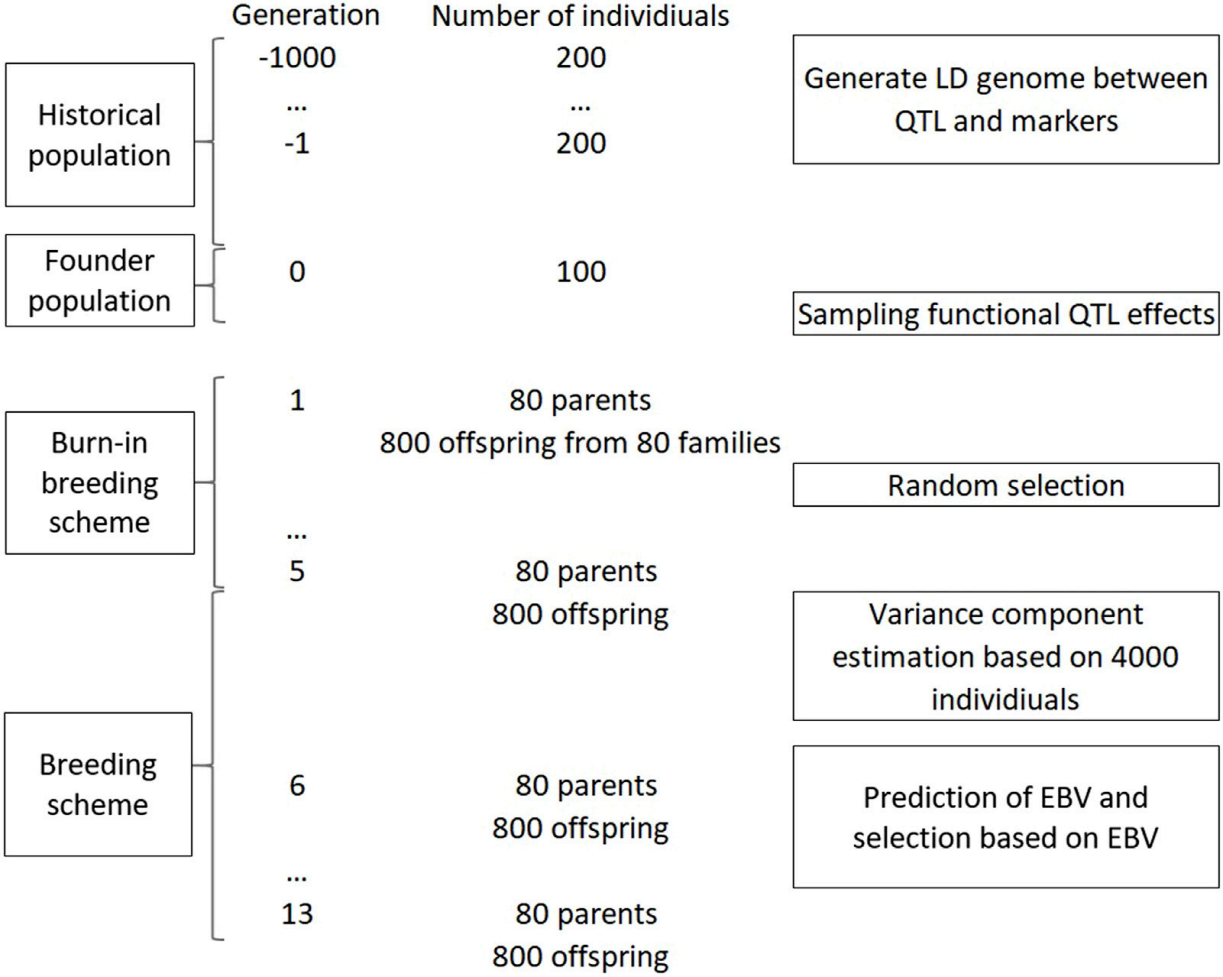
An overview of simulation pipeline in example 1 and 2.

Figure 1 shows the simulation pipeline for this example. The genome of founders was simulated to form LD between QTL and markers using a Fisher-Wright inheritance model (Fisher, 1930). The LD genome consisted of 12 chromosomes with genetic distances emulating that of potato (Massa et al., 2018). The total genome length was 888.6 cM. The initial genome that had 50k marker loci and 10k QTL with an equal frequency for each allele. A historical population with effective population size of 200 was simulated for 1,000 generations of random mating, a simulated bottleneck, and an inheritance pattern of standard Mendelian principles and bivalent chromosome pairing (Voorrips and Maliepaard, 2012). In example 1, 12 founder populations were created corresponding to 6 levels of multi-allelic assumptions and 2 levels of ploidy (Table 3). The 100-individual founders for each of the populations was created, where the genome of these founders consisted of 2k QTL and 10k bi-allelic markers. The QTL and markers were drawn randomly from loci that were segregating with a minor allele frequency ≥0.05. For markers, bi-allelic state was assumed in all scenarios. The percentage of QTL having segerating bi-, tri-, or quad-alleles was corresponding to the assumption of the scenario. In case of multiple allellisms, for example, quad-allelic assumption for a QTL, each of the four alleles must have a minor allele frequency of at least 0.05. The LD pattern in case of bi-allelic loci for diploid and tetraploid genomes can be found in Supplementary Appendix 2. The functional additive variance of the founder populations was simulated at $\sigma_{A}^{2}=1.0$ .

A simplified breeding scheme was simulated for 13 discrete generations. In a generation, 80 parents were crossed, pseudo-randomly with no self-pollination, to create 80 families. A parent could mate any other parents, but each parent could contribute to only maximum of four crosses. Each family had 10 full-sib offspring, thus in total there were 800 offspring per generation. From generation 1 to 5, the 80 parents were randomly selected from the 800 offspring. In generation 6 to 13, the selection of the offspring to be parents in the following generation was based on predicted breeding values. At generation 6, variance components were estimated when the phenotype data consisted of 4,000 individuals. This estimation of variance components ensures that extra variation due to unknown variance components were taken into account. These variance component estimates were used in the models for prediction of breeding values in the subsequent generations. Selection of 80 new parents from 800 individuals were carried out based on the genetic evaluation after the phenotypes in a generation were obtained.

Model Equation 11 were used in example 1 for variance component estimation and prediction of breeding values is as follows:

$$y=\text{Xb}+Z_{u}u+e$$ (11)

where y is the vector of individual phenotypes; b is a vector of the fixed effects of individuals’ generation; u is the vector of breeding values $u∼N\left(0,G_{u}\sigma_{u}^{2}\right)$ , where the relationship matrix for additive genetics ( $G_{u}$ ) was constructed based on bi-allelic markers using method (VanRaden, 2008) for different levels of ploidy assumed in the scenario. The computation of $G_{u}$ was carried out using AGHmatrix R package (Amadeu et al., 2023). X and $Z_{u}$ are design matrices relating individuals to fixed effects and additive genetic effects, respectively. Vector e is an environmental residual term: $e∼N\left(0,I\sigma_{e}^{2}\right)$ , where I is an identity matrix, $\sigma_{e}^{2}$ is the environmental residual variance.

Simulation model Equation 10 is QTL-based whereas Equation 11 is marker-based prediction model. Simulation and prediction models are also different in how the covariate of additive effects is calculated. The covariate in Equation 10 is independent of the allele frequency in the population while the covariate as elements of $G_{u}$ in Equation 11 uses the frequency in calculation (Chu et al., 2024). The variances estimated from Equation 11 are statistical parameters whereas the simulated variances in Equation 10 are functional variances (Chu et al., 2024). However, as non-additive genetic effects were not simulated in this example, the functional and statistical variances in example 1 would be identical.

Each founder population (at generation 0) were replicated 5 times, i.e., a total of 50 replicates were simulated. The breeding scheme at generations 1–13 was replicated 10 times per founder population replicate. Variance component estimation was carried out using REML in the DMUAI module of the DMU package (Madsen and Jensen, 2013). The prediction of breeding values was performed with the DMU4 module of the DMU package. Population accuracy of the predicted breeding values were assessed for the individuals in generation 6. The accuracy was the correlation between true u from the simulated values and predicted $\hat{u}$ in (Equation 11). Rate of genetic gain was assessed as the rate of increase in the genetic mean of population from generation 5 to 13, i.e., rate of genetic gain = $\frac{\bar{u_{13}}-\bar{u_{5}}}{8}$ , where $\bar{u_{5}}$ and $\bar{u_{13}}$ are the genetic means of population at generation 5 and 13.

### 2.4 Example 2

The investigated factors in example 2 included different prediction models for selection, multi-allelic levels and two levels of ploidy (Table 3). This example used four haplotype founder populations from example 1 for scenarios that had multi-allelic assumption of bi-allelic (100%), and bi-allelic (20%) + quad-allelic (80%) QTL at two ploidy levels of diploids and tetraploids. There were three prediction models investigated, thus in total 2 × 2 × 3 = 12 scenarios simulated. The simulation pipeline of this example is the same as in example 1 and Figure 1. However, the simulation model in example 2 included additive, dominance, and epistatic genetic effects, as in (Equation 9). For simulating epistasis, we assumed $n_{ep}=1000$ , with each QTL present in precisely one pair. The functional variance inputs for the simulation model were set for additive $\sigma_{A}^{2}=1.0$ , dominance $\sigma_{D}^{2}=0.25$ , additive × additive $\sigma_{AA}^{2}=0.25$ , additive-dominance $\sigma_{AD}^{2}=0.25$ , dominance × dominance $\sigma_{DD}^{2}=0.25$ , and environmental term $\sigma_{e}^{2}=2$ . The GBLUP models for predicting breeding values included (Equation 11) and two others as follows:

$$y=\text{Xb}+Z_{u}u+Z_{v}v+e$$ (12)

$$y=\text{Xb}+Z_{u}u+Z_{v}v+Z_{\text{uv}}\left(\text{uu}\right)+Z_{\text{uv}}\left(\text{uv}\right)+Z_{\text{vv}}\left(\text{vv}\right)+e$$ (13)

where u is the vector of breeding (additive) values as described in Equation 11; v is the vector of dominance values $v∼N\left(0,G_{v}\sigma_{v}^{2}\right)$ , where $\sigma_{v}^{2}$ is dominance variance, and the relationship matrix for dominance ( $G_{v}$ ) is genomic marker-based, which were calculated using AGHmatrix R package (Amadeu et al., 2023). The construction of dominance relationships between individuals used the method by Vitezica et al. (2017) for diploids, and Endelman et al. (2018) for tetraploids. Vector $\left(\text{uu}\right)$ is additive × additive $\left(\text{uu}\right)∼N\left(0,G_{\text{uu}}\sigma_{uu}^{2}\right)$ , where $\sigma_{uu}^{2}$ is additive × additive variance, and the relationship matrix $G_{\text{uu}}=G_{u}⊙G_{u}$ , where $G_{u}$ is genomic-based relationship as in (Equation 11), ⊙ is the Hadamard product. Similarly, $\left(\text{uv}\right)$ is the vector of additive-dominance: $\left(\text{uv}\right)∼N\left(0,G_{\text{uv}}\sigma_{uv}^{2}\right)$ , where $\sigma_{uv}^{2}$ is the variance, and $G_{\text{uv}}=G_{u}⊙G_{v}$ . Vector $\left(\text{vv}\right)$ is dominance × dominance: $\left(\text{vv}\right)∼N\left(0,G_{\text{vv}}\sigma_{vv}^{2}\right)$ , where $\sigma_{vv}^{2}$ is the variance, and $G_{\text{vv}}=G_{v}⊙G_{v}$ . Other notations and symbols are the same as in (Equation 11). Variances $\sigma_{u}^{2}$ , $\sigma_{v}^{2}$ , $\sigma_{uu}^{2}$ , $\sigma_{uv}^{2}$ and $\sigma_{vv}^{2}$ from Equation 13 are statistical parameters, which are different from functional variances $\sigma_{A}^{2}$ , $\sigma_{D}^{2}$ , $\sigma_{AA}^{2}$ , $\sigma_{AD}^{2}$ and $\sigma_{DD}^{2}$ in Equation 9 (Chu et al., 2024).

Similar to example 1, the breeding scheme at generations 1–13 was replicated for 10 times for each of the five founder population replicates. Variance component estimation and prediction of breeding values were carried out by DMUAI and DMU4 module, respectively, of the DMU package (Madsen and Jensen, 2013). Rate of genetic gain was calculated similar as in example 1, except that the total genetic value $g_{i}$ was used instead of additive values only.

## 3 Results

Table 4 shows genotypic values of additive ( a ) using (Equation 1) and dominance ( d ) using (Equation 3) for diploids and tetraploids when assuming $n_{B}=2$ at a locus with allele $B_{1}$ and $B_{2}$ . With bi-allelic QTL locus of diploids, the genotypic effects of $B_{1}B_{1}$ , $B_{1}B_{2}$ , and $B_{2}B_{2}$ were $-\left(a_{2}-a_{1}\right)$ , 0 and $+\left(a_{2}-a_{1}\right)$ for a , respectively, and 0, $\left(d_{1}+d_{2}\right)$ and 0 for d , respectively. Table 5 shows epistatic genotypic values for diploids due to interactions between loci k and l when assuming $n_{B}=2$ at the two loci. The additive-dominance interaction between the pair of loci k and l is the sum of additive × dominance and dominance × additive interaction effects for the two loci.

**TABLE 4:** Genotypic values of additive ( a ) and dominance ( d ) at one-locus level when assuming bi-allelic QTL.

Genotype	a	d
Diploid		
$B_{1}B_{1}$	$-\left(a_{2}-a_{1}\right)$	0
$B_{1}B_{2}$	0	$\left(d_{1}+d_{2}\right)$
$B_{2}B_{2}$	$\left(a_{2}-a_{1}\right)$	0
Tetraploid		
$B_{1}B_{1}B_{1}B_{1}$	$-\left(a_{2}-a_{1}\right)$	0
$B_{1}B_{1}B_{1}B_{2}$	$-\frac{1}{2}\left(a_{2}-a_{1}\right)$	$\frac{3}{4}\left(d_{1}+d_{2}\right)$
$B_{1}B_{1}B_{2}B_{2}$	0	( $d_{1}+d_{2}$ )
$B_{1}B_{2}B_{2}B_{2}$	$\frac{1}{2}\left(a_{2}-a_{1}\right)$	$\frac{3}{4}\left(d_{1}+d_{2}\right)$
$B_{2}B_{2}B_{2}B_{2}$	$\left(a_{2}-a_{1}\right)$	0

**TABLE 5:** Genotypic values of additive × additive $\left(aa\right)$ , additive - dominance $\left(ad\right)$ , and dominance × dominance $\left(dd\right)$ in two-loci epistatic interactions when assuming bi-allelic QTL.

Genotype	${\left(aa\right)}^{kl}$	${\left(ad\right)}^{kl}$	${\left(dd\right)}^{kl}$
$B_{1}^{k}B_{1}^{k}B_{1}^{l}B_{1}^{l}$	$+\left\{{\left(aa\right)}_{1}^{kl}-{\left(aa\right)}_{2}^{kl}-{\left(aa\right)}_{3}^{kl}+{\left(aa\right)}_{4}^{kl}\right\}$	0	0
$B_{1}^{k}B_{1}^{k}B_{1}^{l}B_{2}^{l}$	0	$+\left\{{\left(ad\right)}_{1}^{kl}-{\left(ad\right)}_{2}^{kl}+{\left(ad\right)}_{3}^{kl}-{\left(ad\right)}_{4}^{kl}\right\}$	0
$B_{1}^{k}B_{1}^{k}B_{2}^{l}B_{2}^{l}$	$-\left\{{\left(aa\right)}_{1}^{kl}-{\left(aa\right)}_{2}^{kl}-{\left(aa\right)}_{3}^{kl}+{\left(aa\right)}_{4}^{kl}\right\}$	0	0
$B_{1}^{k}B_{2}^{k}B_{1}^{l}B_{1}^{l}$	0	$+\left\{{\left(da\right)}_{1}^{kl}-{\left(da\right)}_{2}^{kl}+{\left(da\right)}_{3}^{kl}-{\left(da\right)}_{4}^{kl}\right\}$	0
$B_{1}^{k}B_{2}^{k}B_{1}^{l}B_{2}^{l}$	0	0	${\left(dd\right)}_{1}^{kl}+{\left(dd\right)}_{2}^{kl}+{\left(dd\right)}_{3}^{kl}+{\left(dd\right)}_{4}^{kl}$
$B_{1}^{k}B_{2}^{k}B_{2}^{l}B_{2}^{l}$	0	$-\left\{{\left(da\right)}_{1}^{kl}-{\left(da\right)}_{2}^{kl}+{\left(da\right)}_{3}^{kl}-{\left(da\right)}_{4}^{kl}\right\}$	0
$B_{2}^{k}B_{2}^{k}B_{1}^{l}B_{1}^{l}$	$-\left\{{\left(aa\right)}_{1}^{kl}-{\left(aa\right)}_{2}^{kl}-{\left(aa\right)}_{3}^{kl}+{\left(aa\right)}_{4}^{kl}\right\}$	0	0
$B_{2}^{k}B_{2}^{k}B_{1}^{l}B_{2}^{l}$	0	$-\left\{{\left(ad\right)}_{1}^{kl}-{\left(ad\right)}_{2}^{kl}+{\left(ad\right)}_{3}^{kl}-{\left(ad\right)}_{4}^{kl}\right\}$	0
$B_{2}^{k}B_{2}^{k}B_{2}^{l}B_{2}^{l}$	$+\left\{{\left(aa\right)}_{1}^{kl}-{\left(aa\right)}_{2}^{kl}-{\left(aa\right)}_{3}^{kl}+{\left(aa\right)}_{4}^{kl}\right\}$	0	0

Table 6 shows rate of genetic gains and accuracy of predicted breeding values in a simplified breeding scheme for diploids and tetraploids when different levels of multiple allelism were assumed in example 1. Surprisingly, accuracy of predicted breeding values was not statistically different for altered levels of QTL multi-allelic assumptions, which occurred in the breeding scheme for both diploids and tetraploids. On the contrary, the rate of genetic gains had increasing tendency with increasing levels of multiple allelic QTL in the scheme for tetraploids whereas the genetic gain did not show this tendency in the scheme for diploids. The increasing tendency in genetic gains was most likely due to increased additive variances with higher multiple allelism in tetraploids. The estimated variances for different scenarios in example 1 could be found in Supplementary Table 1, and the true variances at different generations are in Supplementary Table 2.

**TABLE 6:** Genetic gain and accuracy in simulation model with additive effects only of example 1.

Multi-allelic assumption	Diploids		Tetraploids	
	Genetic gain	Accuracy	Genetic gain	Accuracy
Bi-allele (100%)	1.086	0.842	1.068	0.798
Bi-allele (80%) + tri-allele (20%)	1.097	0.837	1.066	0.796
Bi-allele (50%) + tri-allele (50%)	1.074	0.838	1.104	0.807
Bi-allele (80%) + quad-allele (20%)	1.065	0.842	1.061	0.792
Bi-allele (50%) + quad-allele (50%)	1.077	0.844	1.146	0.794
Bi-allele (20%) + quad-allele (80%)	1.088	0.842	1.118	0.795
Standard deviation over replicates in range (for column)	0.044–0.061	0.03–0.06	0.047–0.068	0.04–0.06

Table 7 shows the rate of genetic gains in a breeding scheme where different prediction models were used for selection in example 2. In this example, while the simulation model includes additive, dominance, and epistatic interactions between pairs of loci, different prediction models were used for selection. The prediction models with and without non-additive effects did not lead to statistical differences in rate of genetic gains. In about two thirds of replicates, the model could not estimate epistatic effects (Supplementary Table 3). Different multiple allelism did not lead to a significant change in rate of genetic gains. The variance components estimated from different prediction models in example 2 can be found in Supplementary Table 4, and the true variances of total genetic values at different generations are in Supplementary Table 2.

**TABLE 7:** Genetic gain in simulation model with additive, dominance and epistatic effects of example 2 when different prediction models were used.

Multi-allelic assumption	Prediction model		
	Additive only	Additive and dominance	Additive, dominance and epistasis
Diploids			
Bi-allele (100%)	1.103	1.117	1.114
Bi-allele (20%) + quad-allele (80%)	1.012	0.991	0.994
Tetraploids			
Bi-allele (100%)	1.283	1.275	1.269
Bi-allele (20%) + quad-allele (80%)	1.224	1.238	1.214
Standard deviation over replicates in range (for column)	0.050–0.064	0.048–0.068	0.045–0.065

## 4 Discussion

Our simulation models allow the presence of multi-allelic loci, which is a more realistic assumption for QTL variants. Our simulation model for additive genetic effects is basically the sum of allelic effects. When bi-allelic QTL is assumed, the simulation models applying Equations 1, 3, and 5–7 are identical to the assumption in common genetic textbook, e.g., Falconer and Mackay (1996) and AlphaSimR package (Gaynor et al., 2021). For example, AlphaSimR defines effects of genotype $B_{1}B_{1}$ , $B_{1}B_{2}$ and $B_{2}B_{2}$ as –a , 0 and +a for additive, respectively. These effects would be corresponding to values in Table 4 if a was defined: $a=\left(a_{2}-a_{1}\right)$ . Here, $\left(a_{2}-a_{1}\right)$ is the substitution effect of allele $B_{2}$ for $B_{1}$ , which is also the definition of a in AlphaSimR and Falconer and Mackay (1996). When bi-allelic QTL is assumed, dominance effect in different ploidy levels is based on a digenic dominance model, which is consistent to Gaynor et al. (2021). Similarly, the epistatic effects in Gaynor et al. (2021) are a special case of our simulation model with bi-allelic assumption.

Interestingly, different levels of multi-allelic assumptions for QTL did not affect accuracy of predicted breeding values based on bi-allelic markers in example 1. This may be due to high density of markers and well-structure population where each clone had many full and half-sibs. Multiple markers can link to a given QTL. Therefore, effects of all alleles at the QTL with multi-allelic state could be estimated using bi-allelic markers. For example, three different bi-allelic marker loci that were closely linked to a QTL could combined to code for up to eight different alleles of the QTL. This could be the reason that regardless of possible multiple alleles in QTL, bi-allelic markers with reasonably high density could predict breeding values in many genomic selection programs (Chu et al., 2019; Samorè and Fontanesi, 2016; Hayes et al., 2013).

In example 1, the differences in rate of genetic gain between different degree of multi-allelisms is primarily due to genetic variances. Although the base population variances are simulated as the same values between two populations, existence of multi-allelism can have higher potential variance, or lower loss of genetic variance under selection. For example, selection led to a removal of a “bad” allele at a QTL in the population. The genetic variance due to QTL would be zero in the bi-allelic case, but might be not in the multi-allelic population. However, maintenance of multi-allelic state might require a bigger effective population size. Otherwise, the multi-allelic state could be lost due to random sampling. This could be the explanation for a higher genetic variance of multi-allelic population in case of tetraploids, but not in diploids in example 1.

While the simulation model including additive, dominance and epistasis was the same for scenarios in example 2, different prediction models (Equations 11–13) were employed for selection. Definition of accuracy of prediction is unclear in literature when different prediction models were used in this case, and particularly when multi-allelic QTL was assumed. Therefore, the rate of genetic gains was used as the main criteria to compare prediction models. Surprisingly, the use of different prediction models did not lead to significant changes in the rate of genetic gains. In other words, the use of correct prediction model for selection, i.e., prediction model and simulation model were more similar, did not improve genetic gains of the breeding scheme. The higher level of multi-allelic assumptions for QTL tended to reduce the genetic gains in example 2, which might be due to lower accuracy of prediction. However, the reduction was not significant.

Nonetheless, examples in this paper are small-scale studies to test ADAM-Multi for multi-allelic features. Many other factors that may affect genetic gain, accuracy of predictions and genetic variances in multi-allelic populations include LD between markers and QTL alleles, population structure, population size, and prediction model. Like other software (Gaynor et al., 2021; Pook et al., 2020; Younis et al., 2023), ADAM-Multi uses functional effects for simulating genotypic values of individuals. Functional effects are independent of allele frequency, thus convenient for studying the consequence of selection in breeding programs (Chu et al., 2024). However, the functional effects and variance parameters cannot be obtained directly by model estimation using real data. Therefore, it is difficult to ensure user-defined parameters for the simulated populations. Just like other software (Gaynor et al., 2021; Pook et al., 2020; Younis et al., 2023), ADAM-Multi is still missing an important feature for a transformation between functional and statistical parameters. More theories are needed to be developed for this transformation, particularly, in case of multi-allelic QTL. Nonetheless, with a more realistic assumption of QTL, ADAM-Multi opens research possibility to study the use of genotyping technology of bi-allelic markers, or the need of new genotyping technology to improve accuracy of selection. Particularly, this assumption of QTL remains very relevant for genomic prediction studies involving multiple breeds and populations. For example, different functional effects of QTL could be assumed in two populations, e.g., González-Diéguez et al. (2021).

This paper presented single-trait models, but the program, ADAM-Multi, can be used for simulating multiple traits with different levels of correlations. For example, the scaling and rescaling procedures in simulation of multiple traits use matrix multiplication, inversion and Cholesky decomposition instead of number multiplication, devision and square root calculations as indicated in this paper. Another note is that the number of alleles $n_{B}$ could be defined individually for each of QTL (Supplementary Appendix 1). However, this paper assumes a defined $n_{B}$ for all QTL even when not all alleles in a QTL are segregating. With this assumption, non-segregating alleles’ effects can affect the mean, but the mean can be altered with adding constant values to μ just like in Equation 9. On the contrary, non-segregating alleles do not affect the functional or statistical variances as the frequencies of these alleles are fixed. In addition, additive effects are centered in our simulation models, but the dominance effects are not. This assumption of dominance leads to a positive effect of heterozygous genotypes, as recommended in Wellmann and Bennewitz (2011).

## 5 Conclusion

This paper presented a simulation model capable of simulating genotypic effects generalized for multiple allelic models and different ploidy levels. This model accommodates genotypic effects of additive, dominance, and epistasis. When assuming bi-allelic QTL, the generalized model becomes identical to the model assumption in common simulation programs, and in genetic textbooks. This model is integrated in our software ADAM-Multi.

In a small-scale study, we have shown that with a reasonable density of bi-allelic markers and a well-structured population, genomic models can effectively predict breeding values despite the presence of multi-allelic QTL. It was also shown that the inclusion of non-additive genetic effects in the prediction model for selection did not lead to a significant improvement in the rate of genetic gains of a breeding program.

## Data Availability

The original contributions presented in the study are included in the article/Supplementary Material, further inquiries can be directed to the corresponding author.

## References

[B1] Álvarez-Castro J. M. Carlborg O. R. (2007). A unified model for functional and statistical epistasis and its application in quantitative trait loci analysis. Genetics. 176, 1151–1167. 10.1534/genetics.106.067348 17409082 PMC1894581

[B2] Álvarez-Castro J. M. Crujeiras R. M. (2019). Orthogonal decomposition of the genetic variance for epistatic traits under linkage disequilibrium-applications to the analysis of bateson-dobzhansky-müller incompatibilities and sign epistasis. Front. Genet. 10, 54. 10.3389/fgene.2019.00054 30891057 PMC6411799

[B3] Álvarez-Castro J. M. Yang R.-C. (2011). Multiallelic models of genetic effects and variance decomposition in non-equilibrium populations. Genetica 139, 1119–1134. 10.1007/s10709-011-9614-9 22068562 PMC3247674

[B4] Amadeu R. R. Garcia A. A. F. Munoz P. R. Ferrão L. F. V. (2023). AGHmatrix: genetic relationship matrices in R. Bioinformatics. 39, btad445. 10.1093/bioinformatics/btad445 37471595 PMC10371492

[B5] Bengtsson C. Thomasen J. R. Kargo M. Bouquet A. Slagboom M. (2022). Emphasis on resilience in dairy cattle breeding: possibilities and consequences. J. Dairy Sci. 105, 7588–7599. 10.3168/jds.2021-21049 35863926

[B6] Biová J. Kaňovská I. Chan Y. O. Immadi M. S. Joshi T. Bilyeu K. (2024). Natural and artificial selection of multiple alleles revealed through genomic analyses. Front. Genet. 14, 1320652. 10.3389/fgene.2023.1320652 38259621 PMC10801239

[B7] Chen C. J. Garrick D. Fernando R. Karaman E. Stricker C. Keehan M. (2022). XSim version 2: simulation of modern breeding programs. G3 (Bethesda). 12, jkac032. 10.1093/g3journal/jkac032 35244161 PMC8982375

[B8] Christensen O. F. Madsen P. Nielsen B. Ostersen T. Su G. (2012). Single-step methods for genomic evaluation in pigs. Animal Int. J. Animal Biosci. 6, 1565–1571. 10.1017/s1751731112000742 22717310

[B9] Chu T. T. Bastiaansen J. W. M. Berg P. Romé H. Marois D. Henshall J. (2019). Use of genomic information to exploit genotype-by-environment interactions for body weight of broiler chicken in bio-secure and production environments. Genet. Sel. Evol. 51, 50. 10.1186/s12711-019-0493-3 31533614 PMC6751605

[B10] Chu T. T. Henryon M. Jensen J. Ask B. Christensen O. F. (2021). Statistical model and testing designs to increase response to selection with constrained inbreeding in genomic breeding programs for pigs affected by social genetic effects. Genet. Sel. Evol. 53, 1. 10.1186/s12711-020-00598-8 33397289 PMC7784391

[B11] Chu T. T. Kristensen P. Jensen J. (2024). Simulation of functional additive and non-additive genetic effects using statistical estimates from quantitative genetic models. Hered. (Edinb). 133, 33–42. 10.1038/s41437-024-00690-5 PMC11222558 38822133

[B12] Chu T. T. Sørensen A. C. Lund M. S. Meier K. Nielsen T. Su G. (2020). Phenotypically selective genotyping realizes more genetic gains in a rainbow trout breeding program in the presence of genotype-by-environment interactions. Front. Genet. 866, 866. 10.3389/fgene.2020.00866 PMC7517704 33061932

[B13] Da Y. (2015). Multi-allelic haplotype model based on genetic partition for genomic prediction and variance component estimation using SNP markers. BMC Genet. 16, 144. 10.1186/s12863-015-0301-1 26678438 PMC4683770

[B14] Endelman J. B. Carley C. A. S. Bethke P. C. Coombs J. J. Clough M. E. da Silva W. L. (2018). Genetic variance partitioning and genome-wide prediction with allele dosage information in autotetraploid potato. Genetics. 209, 77–87. 10.1534/genetics.118.300685 29514860 PMC5937173

[B15] Falconer D. S. Mackay T. F. (1996). Introduction to quantitative genetics. England: Pearson Prentice Hall.

[B16] Faux A. M. Gorjanc G. Gaynor R. C. Battagin M. Edwards S. M. Wilson D. L. (2016). AlphaSim: software for breeding program simulation. Plant Genome 9. 10.3835/plantgenome2016.02.0013 27902803

[B17] Fisher R. A. (1930). The genetical theory of natural selection. Oxford: Clarendon.

[B18] Gaynor R. C. Gorjanc G. Hickey J. M. (2021). AlphaSimR: an R package for breeding program simulations. G3 (Bethesda). 11, jkaa017. 10.1093/g3journal/jkaa017 33704430 PMC8022926

[B19] Gebreyesus G. Sahana G. Christian Sørensen A. Lund M. S. Su G. (2020). Novel approach to incorporate information about recessive lethal genes increases the accuracy of genomic prediction for mortality traits. Hered. (Edinb). 125, 155–166. 10.1038/s41437-020-0329-5 PMC7426854 32533106

[B20] González-Diéguez D. Legarra A. Charcosset A. Moreau L. Lehermeier C. Teyssèdre S. (2021). Genomic prediction of hybrid crops allows disentangling dominance and epistasis. Genetics 218, iyab026. 10.1093/genetics/iyab026 33864072 PMC8128411

[B21] Hayes B. J. Lewin H. A. Goddard M. E. (2013). The future of livestock breeding: genomic selection for efficiency, reduced emissions intensity, and adaptation. Trends Genet. 29, 206–214. 10.1016/j.tig.2012.11.009 23261029

[B22] Henryon M. Liu H. Berg P. Su G. Nielsen H. M. Gebregiwergis G. T. (2019). Pedigree relationships to control inbreeding in optimum-contribution selection realise more genetic gain than genomic relationships. Genet. Sel. Evol. 51, 39. 10.1186/s12711-019-0475-5 31286868 PMC6615244

[B23] Henryon M. Ostersen T. Ask B. Sørensen A. C. Berg P. (2015). Most of the long-term genetic gain from optimum-contribution selection can be realised with restrictions imposed during optimisation. Genet. Sel. Evol. 47, 21. 10.1186/s12711-015-0107-7 25887703 PMC4376334

[B24] Jiang Y. Chen S. Wang X. Liu M. Iacono W. G. Hewitt J. K. (2020). Association analysis and meta-analysis of multi-allelic variants for large-scale sequence data. Genes 11, 586. 10.3390/genes11050586 32466134 PMC7288273

[B25] Liu H. Tessema B. B. Jensen J. Cericola F. Andersen J. R. Sørensen A. C. (2019). ADAM-plant: a software for stochastic simulations of plant breeding from molecular to phenotypic level and from simple selection to complex speed breeding programs. Front. Plant Sci. 9, 1926. 10.3389/fpls.2018.01926 30687343 PMC6333911

[B26] Madsen P. Jensen J. (2013). DMU: a user’s guide. A package for analysing multivariate mixed models. Available at: https://dmu.ghpc.au.dk/dmu/DMU/(Accessed September 12, 2021).

[B27] Massa A. N. Manrique-Carpintero N. C. Coombs J. Haynes K. G. Bethke P. C. Brandt T. L. (2018). Linkage analysis and QTL mapping in a tetraploid russet mapping population of potato. BMC Genet. 19, 87. 10.1186/s12863-018-0672-1 30241465 PMC6150958

[B28] Pedersen L. Sørensen A. Henryon M. Ansari-Mahyari S. Berg P. (2009). ADAM: a computer program to simulate selective breeding schemes for animals. Livest. Sci. 121, 343–344. 10.1016/j.livsci.2008.06.028

[B29] Pérez-Enciso M. Ramírez-Ayala L. C. Zingaretti L. M. (2020). SeqBreed: a python tool to evaluate genomic prediction in complex scenarios. Genet. Sel. Evol. GSE 52, 7. 10.1186/s12711-020-0530-2 32039696 PMC7008576

[B30] Pook T. Schlather M. Simianer H. (2020). MoBPS - modular breeding program simulator. G3 Genes. Genomes. Genetics 10, 1915–1918. 10.1534/g3.120.401193 32229505 PMC7263682

[B31] Romé H. Chu T. T. Marois D. Huang C.-H. Madsen P. Jensen J. (2023). Estimation and consequences of direct-maternal genetic and environmental covariances in models for genetic evaluation in broilers. Genet. Sel. Evol. 55, 58. 10.1186/s12711-023-00829-8 37550635 PMC10405509

[B32] Samorè A. B. Fontanesi L. (2016). Genomic selection in pigs: state of the art and perspectives. Ital. J. Anim. Sci. 15, 211–232. 10.1080/1828051X.2016.1172034

[B33] Sargolzaei M. Schenkel F. S. (2009). QMSim: a large-scale genome simulator for livestock. Bioinformatics. 25, 680–681. 10.1093/bioinformatics/btp045 19176551

[B34] Tessema B. B. Liu H. Sørensen A. C. Andersen J. R. Jensen J. (2020). Strategies using genomic selection to increase genetic gain in breeding programs for wheat. Front. Genet. 11, 578123. 10.3389/fgene.2020.578123 33343626 PMC7748061

[B35] Thérèse Navarro A. Tumino G. Voorrips R. E. Arens P. Smulders M. J. M. van de Weg E. (2022). Multiallelic models for QTL mapping in diverse polyploid populations. BMC Bioinforma. 23, 67. 10.1186/s12859-022-04607-z PMC8842866 35164669

[B36] VanRaden P. M. (2008). Efficient methods to compute genomic predictions. J. Dairy Sci. 91, 4414–4423. 10.3168/jds.2007-0980 18946147

[B37] Vitezica Z. G. Legarra A. Toro M. A. Varona L. (2017). Orthogonal estimates of variances for additive, dominance, and epistatic effects in populations. Dominance, Epistatic Eff. Populations 206, 1297–1307. 10.1534/genetics.116.199406 PMC5500131 28522540

[B38] Voorrips R. E. Maliepaard C. A. (2012). The simulation of meiosis in diploid and tetraploid organisms using various genetic models. BMC Bioinforma. 13, 248. 10.1186/1471-2105-13-248 PMC3542247 23013469

[B39] Weber S. E. Frisch M. Snowdon R. J. Voss-Fels K. P. (2023). Haplotype blocks for genomic prediction: a comparative evaluation in multiple crop datasets. Front. plant Sci. 14, 1217589. 10.3389/fpls.2023.1217589 37731980 PMC10507710

[B40] Wellmann R. Bennewitz J. (2011). The contribution of dominance to the understanding of quantitative genetic variation. Genet. Res. 93, 139–154. 10.1017/S0016672310000649 21481291

[B41] Yang R.-C. Álvarez-Castro J. M. (2008). Functional and statistical genetic effects with multiple alleles.

[B42] Younis O. G. Turchetta M. Ariza Suarez D. Yates S. Studer B. Athanasiadis I. N. (2023). ChromaX: a fast and scalable breeding program simulator. Bioinformatics 39, btad691. 10.1093/bioinformatics/btad691 37991849 PMC10709540

[B43] Zaalberg R. M. Villumsen T. M. Jensen J. Chu T. T. (2022). Effective selection for lower mortality in organic pigs through selection for total number born and number of dead piglets. Animals. 12, 1796. 10.3390/ani12141796 35883342 PMC9311777

