# Supplementary material for: ADAM-multi: software to simulate complex breeding programs for animals and plants with different ploidy levels and generalized genotypic effect models to account for multiple alleles
Source: Front Genet. 2025 Feb 10;16:1513615. doi: 10.3389/fgene.2025.1513615 (PMC11847855; doi:10.3389/fgene.2025.1513615)
Supplement: Supplementary file 1 [file Table1.docx]

Supplementary Material

**Appendix 1: Genotypic models in case of arbitrary number of alleles for different QTL**

In the main paper, we show the genotypic models for simulation in the case that number ($n_{B}$) of alleles are assumed the same for all QTL even if not all alleles in a QTL are segregating. Here, we show the genotypic models in the case of arbitrary number of alleles for different QTL.

The additive genotypic value ($a$) at locus $x$ is:

$a^{x}=\sum_{i_{B}}^{n_{B}^{x}} t_{i_{B}}^{a,x}a_{i_{B}}^{x}=t_{1}^{a,x}a_{1}^{x}+t_{2}^{a,x}a_{2}^{x}+\ldots+t_{n_{B}^{x}}^{a,x}a_{n_{B}^{x}}^{x}$ (Eq. S1)

where $n_{B}^{x}$ is the number of segregating alleles for QTL $k$; $t_{i_{B}}^{a,x}$ is calculated as Eq. 2 in the main paper for locus $x$. Eq. S1 is equivalent to Eq. 1 in the main paper.

The dominance genotypic value ($d$) at locus $x$ that is equivalent to Eq. 3 in the main paper is:

$d^{x}=\sum_{i_{B}}^{n_{B}^{x}} t_{i_{B}}^{d,x}d_{i_{B}}^{x}=t_{1}^{d,x}d_{1}^{x}+t_{2}^{d,x}d_{2}^{x}+\ldots+t_{n_{B}^{x}}^{d,x}d_{n_{B}^{x}}^{x}$ (Eq. S2)

where $t_{i_{B}}^{d,x}$ is calculated as Eq. 4 in the main paper for locus $x$.

The additive × additive genotypic value ${(aa)}^{kl}$ of the epistatic interaction between the pair of loci $k$ and $l$ is:

${(aa)}^{kl}=\left\{ \left[ \begin{matrix} t_{1}^{a,l} & t_{2}^{a,l} & \ldots& t_{n_{B}^{l}}^{a,l} \end{matrix} \right]\otimes\left[ \begin{matrix} t_{1}^{a,k} & t_{2}^{a,k} & \ldots& t_{n_{B}^{k}}^{a,k} \end{matrix} \right] \right\}\cdot\left[ \begin{matrix} \left( aa \right)_{1}^{kl} \\ \left( aa \right)_{2}^{kl} \\ \ldots\\ \left( aa \right)_{n_{B}^{k}\times n_{B}^{l}}^{kl} \end{matrix} \right]$ (Eq. S3)

where $n_{B}^{k}$ and $n_{B}^{l}$ is the number of segregating alleles for QTL $k$ and $l$, respectively.

The additive-dominance genotypic value ${(ad)}^{kl}$ of the epistatic interaction between the pair of loci $k$ and $l$ is:

${(ad)}^{kl}=\left\{ \left[ \begin{matrix} t_{1}^{d,l} & t_{2}^{d,l} & \ldots& t_{n_{B}^{l}}^{d,l} \end{matrix} \right]\otimes\left[ \begin{matrix} t_{1}^{a,k} & t_{2}^{a,k} & \ldots& t_{n_{B}^{k}}^{a,k} \end{matrix} \right] \right\}\cdot\left[ \begin{matrix} \left( ad \right)_{1}^{kl} \\ \left( ad \right)_{2}^{kl} \\ \ldots\\ \left( ad \right)_{n_{B}^{k}\times n_{B}^{l}}^{kl} \end{matrix} \right]+\left\{ \left[ \begin{matrix} t_{1}^{a,l} & t_{2}^{a,l} & \ldots& t_{n_{B}^{l}}^{a,l} \end{matrix} \right]\otimes\left[ \begin{matrix} t_{1}^{d,k} & t_{2}^{d,k} & \ldots& t_{n_{B}^{k}}^{d,k} \end{matrix} \right] \right\}\cdot\left[ \begin{matrix} \left( da \right)_{1}^{kl} \\ \left( da \right)_{2}^{kl} \\ \ldots\\ \left( da \right)_{n_{B}^{k}\times n_{B}^{l}}^{kl} \end{matrix} \right]$ (Eq. S4)

The dominance × dominance genotypic value ${(dd)}^{kl}$ of the epistatic interaction between the pair of loci $k$ and $l$:

${(dd)}^{kl}=\left\{ \left[ \begin{matrix} t_{1}^{d,l} & t_{2}^{d,l} & \ldots& t_{n_{B}^{l}}^{d,l} \end{matrix} \right]\otimes\left[ \begin{matrix} t_{1}^{d,k} & t_{2}^{d,k} & \ldots& t_{n_{B}^{k}}^{d,k} \end{matrix} \right] \right\}\cdot\left[ \begin{matrix} \left( dd \right)_{1}^{kl} \\ \left( dd \right)_{2}^{kl} \\ \ldots\\ \left( dd \right)_{n_{B}^{k}\times n_{B}^{l}}^{kl} \end{matrix} \right]$ (Eq. S5)

Equations Eq. S3, S4, and S5 are equivalent to Eq. 5, 6, and 7 in the main paper, respectively.

Simulation of the total genotypic value of the individual level could be set up as the sum effects that are due to all QTLs and epistatic pair interactions. Here, we present the formula in matrix form that is set up to be equivalent to Eq. 8 in the main paper. The formula for total genotypic value $g_{i}$ of individual $i$ is:

$g_{i}=\mathrm{tr}\left( \mathbf{t}_{\mathbf{i}}^{\mathbf{a}}\cdot\mathbf{a} \right)+\mathrm{tr}\left( \mathbf{t}_{\mathbf{i}}^{\mathbf{d}}\cdot\mathbf{d} \right)+tr[\mathbf{t}_{\mathbf{i}}^{\mathbf{aa}}\cdot\left( \mathbf{aa} \right)]+tr[\mathbf{t}_{\mathbf{i}}^{\mathbf{dd}}\cdot\left( \mathbf{dd} \right)]+tr[\mathbf{t}_{\mathbf{i}}^{\mathbf{ad}}\cdot\left( \mathbf{ad} \right)]+tr[\mathbf{t}_{\mathbf{i}}^{\mathbf{da}}\cdot\left( \mathbf{da} \right)]$ (Eq. S6)

where $\mathbf{a}$ is a $n_{B}\times n_{qtl}$ matrix, where $n_{B}$ is the maximum number of segregating alleles for all QTL.

$\mathbf{a=}\left[ \begin{matrix} a_{1}^{j_{1}} & a_{1}^{j_{2}} & \ldots& a_{1}^{n_{qtl}} \\ a_{2}^{j_{1}} & a_{2}^{j_{2}} & \ldots& a_{2}^{n_{qtl}} \\ \ldots& \ldots& \ldots& \ldots\\ a_{n_{B}^{1}}^{j_{1}} & a_{n_{B}^{2}}^{j_{2}} & \ldots& a_{n_{B}^{n_{qtl}}}^{n_{qtl}} \\ a_{n_{B}^{1}+1}^{0} & a_{n_{B}^{2}+1}^{0} & \ldots& a_{n_{B}^{n_{qtl}}+1}^{0} \\ \ldots& \ldots& \ldots& \ldots\\ a_{n_{B}}^{0} & a_{n_{B}}^{0} & \ldots& a_{n_{B}}^{0} \end{matrix} \right]$, where $n_{qtl}$ is the number of QTL; $a_{i_{B}}^{j_{qtl}}$ is the additive genotypic effect of allele $i_{B}^{j_{qtl}}$ at locus $j_{qtl}$; $n_{B}^{j_{qtl}}$is the number of segregating alleles at locus $j_{qtl}$. The maximum of $n_{B}^{j_{qtl}}$ is $n_{B}$. If $n_{B}^{j_{qtl}}=n_{B}$, $a_{n_{B}^{j_{qtl}}}^{j_{qtl}}=a_{n_{B}}^{j_{qtl}}$. If $n_{B}^{j_{qtl}}<n_{B}$, the additive genotypic effects of non-segregating alleles from $(n_{B}^{j_{qtl}}+1)$ to $n_{B}$ are set at zero, or $\left[ \begin{matrix} a_{n_{B}^{j_{qtl}}+1}^{0} & \ldots& a_{n_{B}}^{0} \end{matrix} \right]^{'}=\left[ \begin{matrix} 0 & \ldots& 0 \end{matrix} \right]^{'}$. Matrix $\mathbf{t}_{\mathbf{i}}^{\mathbf{a}}$ with a dimension of $n_{qtl}$ rows and $n_{B}$ columns is set up in the same way as in Eq. 8 the main paper. Similarly, $\mathbf{d}$ is a $n_{B}\times n_{qtl}$ matrix of dominance genotypic effects; $\mathbf{t}_{\mathbf{i}}^{\mathbf{d}}$ is a $n_{qtl}\times n_{B}$ matrix of dominance covariate of individual $i$.

Matrix $\left( \mathbf{aa} \right)$ has a dimension of $\left( n_{B}\times n_{B} \right)$ rows and $n_{ep}$ columns. Column $j_{ep}$ of $\left( \mathbf{aa} \right)$ is vector $\left( \mathbf{aa} \right)_{j_{ep}}$:

$\left( \mathbf{aa} \right)_{j_{ep}}\boldsymbol{=}\left[ \begin{matrix} \left( aa \right)_{1}^{kl} & \left( aa \right)_{2}^{kl} & \ldots& \left( aa \right)_{n_{B}^{l}}^{kl} & \left( aa \right)_{n_{B}^{l}+1}^{0} & \ldots& \left( aa \right)_{n_{B}}^{0} \end{matrix} \right]^{'}\otimes{\mathbf{J}_{\boldsymbol{k}}}^{\boldsymbol{'}}$,

Where $\left( aa \right)_{i_{B}^{l}}^{kl}$ are the epistatic additive × additive effects at the interaction pair $j_{ep}$. If $n_{B}^{l}<n_{B}$, $\left[ \begin{matrix} \left( aa \right)_{n_{B}^{l}+1}^{0} & \ldots& \left( aa \right)_{n_{B}}^{0} \end{matrix} \right]=\left[ \begin{matrix} 0 & \ldots& 0 \end{matrix} \right]$. Vector $\mathbf{J}_{\boldsymbol{k}}$ with a dimension of $n_{B}$ row and $1$ columns is a positioning matrix. Elements of $\mathbf{J}_{\boldsymbol{k}}$ from 1^st^ to ${n_{B}^{k}}^{th}$ are one. If $n_{B}^{k}<n_{B}$, the elements from ${(n_{B}^{k}+1)}^{th}$ to $n_{B}$ are zero. So, ${\mathbf{J}_{\boldsymbol{k}}}^{\boldsymbol{'}}\boldsymbol{=}\left[ \begin{matrix} 1_{1} & 1_{2} & \ldots& 1_{n_{B}^{k}} & 0_{n_{B}^{k}+1} & \ldots& 0_{n_{B}} \end{matrix} \right]^{'}$ . Similarly, $\left( \mathbf{ad} \right)$, $\left( \mathbf{d}\mathbf{a} \right)$ and$\left( \mathbf{dd} \right)$ are $(n_{B}\times n_{B})\times n_{ep}$ matrices of additive × dominance, dominance × additive and dominance × dominance genotypic effects. These matrices are set up the similar way as for $\left( \mathbf{aa} \right)$. Matrices $\mathbf{t}_{\mathbf{i}}^{\mathbf{aa}}$, $\mathbf{t}_{\mathbf{i}}^{\mathbf{ad}}$, $\mathbf{t}_{\mathbf{i}}^{\mathbf{d}\mathbf{a}}$ and $\mathbf{t}_{\mathbf{i}}^{\mathbf{dd}}$ in Eq. S6 is set up in the same way as in Eq. 8 the main paper.

**Appendix 2: Linkage disequilibrium (LD) for simulated diploid and tetraploid genomes of potato**

The LD measure $r^{2}$ was calculated for diploid and tetraploid genomes using R package ‘ldsep’ (Gerard, 2021). These calculations were done for adjacent loci pairs, and loci pairs within maximum of 5cM genetic distance for LD decay pattern. Note that genetic and physical distances between loci are about equivalent for potato, eg. 1 centiMorgan (cM) ~ 1 Mega base pair (Mbp).

***Appendix 2.1. Summary statistics of pairwise LD between adjacent loci***

|  | $r^{2}$ | | Genetic distance (cM) | |
| --- | --- | --- | --- | --- |
|  | Diploids | Tetraploids | Diploids | Tetraploids |
| Mean | 0.555 | 0.298 | 0.074 | 0.074 |
| SD | 0.366 | 0.313 | 0.128 | 0.080 |
| Min | 0.0 | 0.0 | 0.0 | 0.0 |
| Max | 0.961 | 0.98 | 2.000 | 0.839 |


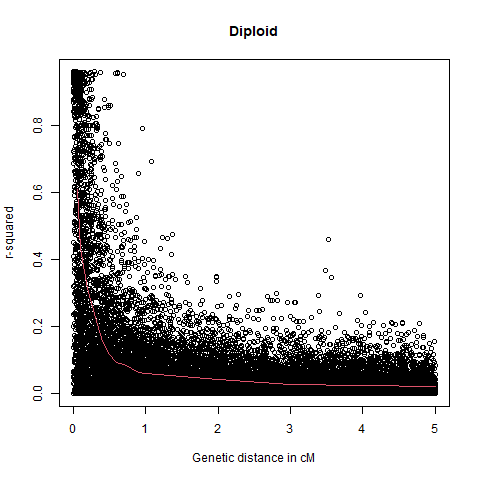

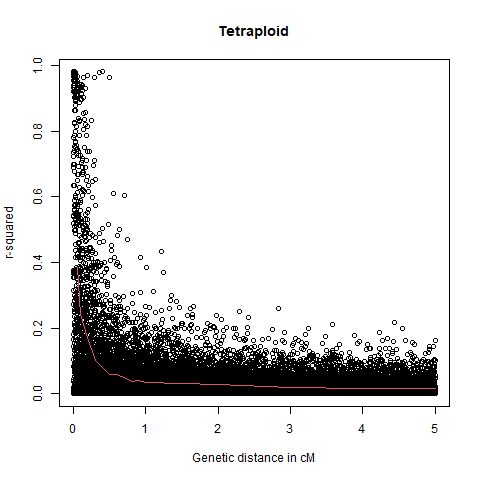


***Appendix 2.2. LD decays of pairwise*** $\boldsymbol{r}^{\boldsymbol{2}}$ ***versus genetic distance (cM)***

Gerard, D., (2021). Pairwise linkage disequilibrium estimation for polyploids. Molecular Ecology Resources. 21, 1230-1242.

**Supplementary Table 1: Variance components estimated from prediction model in example 1**

| Ploidy | Diploids | | Tetraploids | |
| --- | --- | --- | --- | --- |
| Multi-allelic assumption | Additive | Residual | Additive | Residual |
| Simulated values | 1.0 | 2.0 | 1.0 | 2.0 |
| Bi-allele (100%) | 0.980 | 2.026 | 0.911 | 2.028 |
| Bi-allele (80%) + tri-allele (20%) | 0.973 | 2.014 | 0.920 | 2.028 |
| Bi-allele (50%) + tri-allele (50%) | 0.959 | 2.006 | 0.944 | 2.032 |
| Bi-allele (80%) + quad-allele (20%) | 0.963 | 2.008 | 0.902 | 2.031 |
| Bi-allele (50%) + quad-allele (50%) | 0.989 | 2.026 | 0.963 | 2.048 |
| Bi-allele (20%) + quad-allele (80%) | 0.956 | 2.020 | 0.954 | 2.048 |
| Standard deviation over replicates in range (for column) | 0.063-0.087 | 0.048-0.057 | 0.084-0.095 | 0.051-0.060 |

**Supplementary Table 2: True total genetic variance of population at generation 5, 10 and 13 in example 1**

| Multi-allelic assumption | Generation 5 | | Generation 10 | | Generation 13 | |
| --- | --- | --- | --- | --- | --- | --- |
|  | Diploids | Tetraploids | Diploids | Tetraploids | Diploids | Tetraploids |
| Bi-allele (100%) | 0.995 | 0.960 | 0.522 | 0.590 | 0.402 | 0.531 |
| Bi-allele (80%) + tri-allele (20%) | 0.987 | 0.976 | 0.534 | 0.576 | 0.404 | 0.537 |
| Bi-allele (50%) + tri-allele (50%) | 0.953 | 0.986 | 0.488 | 0.615 | 0.400 | 0.576 |
| Bi-allele (80%) + quad-allele (20%) | 0.969 | 0.947 | 0.486 | 0.585 | 0.378 | 0.527 |
| Bi-allele (50%) + quad-allele (50%) | 0.986 | 0.998 | 0.486 | 0.663 | 0.374 | 0.580 |
| Bi-allele (20%) + quad-allele (80%) | 0.973 | 0.980 | 0.500 | 0.623 | 0.396 | 0.565 |
| Standard deviation over replicates in range (for column) | 0.069- 0.091 | 0.074- 0.092 | 0.051- 0.066 | 0.044- 0.058 | 0.035- 0.047 | 0.046- 0.057 |

**Supplementary Table 3: Number of replicates that epistasis could be estimated in prediction model with additive, dominance and epistasis in example 2**

| Ploidy | Diploids |  | Tetraploids |  |
| --- | --- | --- | --- | --- |
| Multi-allelic assumption | # well-converged | # non-converged | # well-converged | # non-converged |
| Bi-allele (100%) | 16 | 34 | 18 | 32 |
| Bi-allele (20%) + quad-allele (80%) | 14 | 36 | 30 | 20 |

Note: In non-converged replicate, prediction of breeding values in the sequential generation used the variance component values from the previous converged replicate.

**Supplementary Table 4: Variance components estimated from different prediction models in example 2, excluding non-converged replicates**

| Variance | Bi-allele (100%) | | | Bi-allele (20%) + quad-allele (80%) | | |
| --- | --- | --- | --- | --- | --- | --- |
|  | U | U+V | Full | U | U+V | Full |
| Diploids |  |  |  |  |  |  |
| Additive | 1.562 | 1.505 | 1.369 | 1.471 | 1.372 | 1.285 |
| Dominance |  | 0.418 | 0.372 |  | 0.528 | 0.490 |
| Additive × additive |  |  | 0.257 |  |  | 0.185 |
| Additive-dominance |  |  | 0.098 |  |  | 0.087 |
| Dominance × dominance |  |  | 0.267 |  |  | 0.171 |
| Residual | 2.613 | 2.220 | 1.711 | 2.637 | 2.132 | 1.795 |
| Tetraploids |  |  |  |  |  |  |
| Additive | 1.679 | 1.594 | 1.505 | 1.673 | 1.610 | 1.499 |
| Dominance |  | 0.287 | 0.216 |  | 0.306 | 0.229 |
| Additive × additive |  |  | 0.287 |  |  | 0.287 |
| Additive-dominance |  |  | 0.203 |  |  | 0.128 |
| Dominance × dominance |  |  | 0.263 |  |  | 0.277 |
| Residual | 2.393 | 2.175 | 1.588 | 2.330 | 2.088 | 1.568 |
| Standard deviation over replicates in range (for column) | 0.075- 0.168 | 0.048- 0.156 | 0.051- 0.226 | 0.070- 0.167 | 0.058- 0.135 | 0.044- 0.285 |

Note:

Prediction model U refers to PM. 1 in the main paper, where the model included additive genetic effects only

Prediction model U+V refers to PM. 2 that included additive and dominance effects

Prediction model Full refers to PM. 3 that included additive, dominance, and epistatic effects

Functional variances of the simulation model in the founder population are 1.0, 0.25, 0.25, 0.25, 0.25 and 2.0 for additive, dominance, additive × additive, additive-dominance, dominance × dominance and environmental term, respectively.

**Supplementary Table 5: True total genetic variance of population at generation 5, 10 and 13 in example 2**

| Multi-allelic assumption | Generation 5 | | | Generation 10 | | | Generation 13 | | |
| --- | --- | --- | --- | --- | --- | --- | --- | --- | --- |
|  | U | U+V | Full | U | U+V | Full | U | U+V | Full |
| Diploids |  |  |  |  |  |  |  |  |  |
| Bi-allele (100%) | 2.143 | 2.096 | 2.080 | 1.521 | 1.472 | 1.531 | 1.289 | 1.310 | 1.298 |
| Bi-allele (20%) + quad-allele (80%) | 2.051 | 2.075 | 2.113 | 1.524 | 1.523 | 1.505 | 1.406 | 1.333 | 1.360 |
| Tetraploids |  |  |  |  |  |  |  |  |  |
| Bi-allele (100%) | 2.053 | 2.057 | 2.083 | 1.417 | 1.433 | 1.430 | 1.306 | 1.327 | 1.320 |
| Bi-allele (20%) + quad-allele (80%) | 2.022 | 2.022 | 2.005 | 1.359 | 1.379 | 1.382 | 1.271 | 1.320 | 1.280 |
| Standard deviation over replicates in range (for column) | 0.108- 0.151 | 0.105- 0.145 | 0.117- 0.144 | 0.109- 0.158 | 0.108- 0.148 | 0.087- 0.165 | 0.077- 0.159 | 0.122- 0.148 | 0.092- 0.135 |

Note:

Prediction model U refers to PM. 1 in the main paper, where the model included additive genetic effects only

Prediction model U+V refers to PM. 2 that included additive and dominance effects

Prediction model Full refers to PM. 3 that included additive, dominance, and epistatic effects
